# Temporal trend in venous thromboembolism hospitalization rates in Brazil

**DOI:** 10.1590/1806-9282.20240608

**Published:** 2025-03-31

**Authors:** Felipe Soares Oliveira Portela, Nelson Wolosker, Andressa Cristina Sposato Louzada, Maria Fernanda Cassino Portugal, Marcelo Fiorelli Alexandrino da Silva, Diogo Lopes Cintra, Giulia de Payrebrune St Sève Marins Girardi, Alexandre Fioranelli, Marcelo Passos Teivelis

**Affiliations:** 1 Hospital Israelita Albert Einstein, Department of Vascular Surgery – São Paulo (SP), Brazil.; 2 Faculdade Israelita de Ciências da Saúde Albert Einstein – São Paulo (SP), Brazil.

**Keywords:** Venous thromboembolism, Pulmonary embolism, Venous thrombosis, Big data, Public health

## Abstract

**OBJECTIVE:**

Venous thromboembolism is a condition of great interest to public health, as it is potentially preventable and has a high morbidity and mortality potential. Knowing the real-world data in a country of continental dimensions such as Brazil is essential to help define health policies that enable proper diagnosis and treatment of this disease. The objective of this study was to evaluate the incidence and in-hospital mortality rates of venous thromboembolism in public hospitals under Brazil's public health system.

**METHODS:**

This is a population-based, cross-sectional, retrospective analysis of all hospitalizations for venous thromboembolism in the Brazilian public health system between 2008 and 2022. Using a public database, all hospital admissions for thromboembolic events were selected, defining the incidence, in-hospital mortality, and differences between Brazilian macro-regions.

**RESULTS:**

A total of 700,315 admissions for venous thromboembolism were documented in the Brazilian public health system between 2008 and 2022, which represents 3.02 admissions per 10,000 inhabitants per year. The Southeast region accounted for more than half (54.5%) of the hospitalizations. The highest incidence of hospitalizations occurred in the wealthiest regions (Southeast and South), while the lowest incidence was observed in the poorest regions (North and Northeast). On the other hand, a higher proportion of in-hospital mortality was observed in the North and Northeast regions.

**CONCLUSION:**

The highest admission rates were registered in wealthier regions, while a higher proportion of deaths was found in the poorer ones. This may reveal the difficulty in accessing healthcare services in the North and Northeast regions, which is reflected in the potential underdiagnosis of thromboembolic events in these regions.

## INTRODUCTION

Venous thromboembolism (VTE), which encompasses deep vein thrombosis (DVT) and pulmonary embolism (PE), is a potentially preventable and fatal disease, representing a major public health concern^1,2^.

Population-based and nationwide surveillance systems are the gold standard for determining VTE's true incidence^3^. There are some nationwide VTE epidemiological studies, but their data are highly variable, with VTE incidence ranging from 79 per 100,000 population in Hong Kong^4^ to 269 per 100,000 population in Denmark^5^, making it difficult to set health policies based solely on the epidemiology of different countries.

In addition to variation in incidence, existing nationwide reports are from high-income countries, especially from Western Europe, North America, and East Asia, which may decrease external validity due to possible differences in access to healthcare services and prevalence of risk factors, such as differences in the prevalence of some cancers that pose a higher risk of VTE, the proportion of pregnant women, the incidence of trauma, and the ethnicity proportion of the populations^3,6^.

Brazil is a country of continental dimensions, with most Brazilians identifying themselves as African descendants^7^. As far as we know, there is no nationwide study on the epidemiology of VTE in a middle-income Latin American country, nor with a large proportion of African descendants.

Therefore, we designed this research to study the incidence of VTE in public hospitals under Brazil's public health system (Sistema Único de Saúde), which exclusively insures about 160 million Brazilians, and the trends of VTE by geographic region between 2008 and 2022. The secondary objectives were to evaluate in-hospital mortality rates for PE during the study period to assess whether they showed related trends.

## METHODS

This is a retrospective ecological descriptive study analyzing data available from the digital platform of the Brazilian Public Health System's Department of Informatics of the Unified Health System (DATASUS)^8^, which provides open data on procedures performed by the Brazilian public healthcare system. The platform offers only unidentified data; for this reason, the application of informed consent was not feasible, and it was, therefore, waived by the Institutional Ethics Committee.

All hospitalizations between January 2008 and December 2022 listed under the International Classification of Diseases 10th ed. codes I26 (PE), I80 (phlebitis and thrombophlebitis), and I82 (other venous embolism and thrombosis) were included.

All information was retrieved from a publicly available platform using the web scraping software. Coding was performed in Python v. 2.7.13 (Python Software Foundation, Beaverton, OR, USA) running on Windows 10 Single Language operating system (Microsoft Corporation, Redmond, WA, USA). Data collection, field selection, and table sorting were performed using open-source packages such as Selenium Web Driver v. 3.1.8 (Selenium HQ, with various developers worldwide) and pandas v. 2.7.13 (Lambda Foundry, Inc. and PyData Development Team, NY, USA).

Following data collection, standard data transformation and cleaning procedures were performed for each file, including removing header and footer information, removing the health facility code, and converting date columns to rows. All data were saved and stored in a Microsoft Office Excel 2016^®^ v. 16.0.4456.1003 spreadsheet (Microsoft Corporation). The following information was extracted from the platform: total hospitalizations and in-hospital all-cause mortality per geographic region.

The calculation of incidences was carried out considering only the portion of the Brazilian population that depends exclusively on the public health system (around 75% of the total population). As there is no data available for private healthcare services (around 25% of the country's population), using the total population would distort the results.

Population data were retrieved from the electronic address of the Brazilian Institute for Geography and Statistics (IBGE)^7^.

## RESULTS

Between 2008 and 2022, a total of 700,315 hospitalizations due to VTE were documented. A total of 114,247 (16.3%) hospitalizations were coded I26 (PE), 539,232 (77%) were coded I80 (phlebitis and thrombophlebitis), and 46,836 (6.7%) hospital admissions were coded I82 (other venous embolism and thrombosis).

The yearly average was 46,688 cases, a total of 3.02 cases per 10,000 inhabitants per year, considering the mean population dependent on the public health system between 2008 and 2022.

The distribution of cases by year and geographical region is shown in Table 1. More than half of Brazil's hospital admissions for VTE occurred in the Southeast, the most populous region. The relative frequencies of each region remained relatively stable, but while the Northeast region had an increase of almost 6% (10.57% of the total hospital admissions in 2008 vs. 16.05% in 2022), the South region had a percentage decrease of the same magnitude (26.48% in 2008 vs. 20.92% in 2022).

**Table 1:** Absolute and relative frequencies of admissions due to venous thromboembolism according to geographical region in Brazil, from 2008 to 2022.

Year	North		Northeast		Southeast		South		Center-West	
	n	%	n	%	n	%	n	%	n	%
2008	792	2.39	3,499	10.57	17,759	53.63	8,770	26.48	2,294	6.93
2009	825	2.17	4,283	11.28	20,771	54.71	9,594	25.27	2,492	6.56
2010	891	2.10	4,787	11.27	23,624	55.63	10,395	24.48	2,766	6.51
2011	952	2.15	5,583	12.60	23,962	54.07	10,693	24.13	3,130	7.06
2012	1,005	2.18	6,152	13.33	25,059	54.31	11,068	23.99	2,853	6.18
2013	1,000	2.12	6,624	14.02	25,545	54.05	11,206	23.71	2,888	6.11
2014	1,051	2.25	6,568	14.06	25,307	54.18	11,025	23.60	2,757	5.90
2015	1,095	2.27	6,916	14.32	25,922	53.69	11,505	23.83	2,847	5.90
2016	1,150	2.38	6,782	14.01	26,525	54.79	11,136	23.00	2,822	5.83
2017	1,087	2.27	6,886	14.36	26,332	54.93	10,968	22.88	2,665	5.56
2018	1,127	2.18	7,367	14.25	28,919	55.95	11,480	22.21	2,793	5.40
2019	1,331	2.41	8,588	15.57	30,314	54.97	11,880	21.54	3,034	5.50
2020	1,215	2.50	7,715	15.90	26,344	54.29	10,356	21.34	2,893	5.96
2021	1,304	2.64	8,202	16.61	27,099	54.88	9,717	19.68	3,058	6.19
2022	1,644	3.10	8,501	16.05	28,276	53.38	11,081	20.92	3,469	6.55
Total	16,469	2.34	98,453	13.88	381,758	54.50	160,874	23.14	42,761	6.14

**Figure 1:**
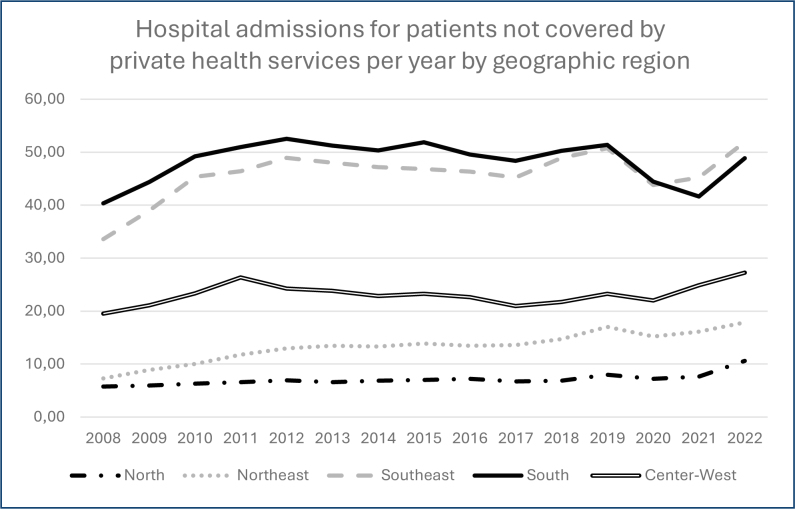
Hospital admissions (per 100,000 inhabitants) for patients not covered by private healthcare services per year by geographic region.

Yearly hospitalization rates per 100,000 inhabitants by geographical region are shown in Figure 1. The Southeast and South regions presented the highest incidence of hospital admissions during the whole period of study.

The average incidence of hospitalizations by age group over the period of 15 years shows that hospitalizations for VTE increase with age, ranging from approximately 4 cases per million inhabitants per year in children aged 0–9 years to approximately 230 cases in the adult population aged 35–44 years and reaching almost 1,000 hospitalizations per million inhabitants per year in people over 80 years of age.

A total of 36,742 in-hospital deaths occurred from DVT hospitalizations during the study period; 5.25% of hospital admissions for VTE evolved with in-hospital deaths. This is detailed in Table 2. The Northeast region had the highest mortality rate in relation to hospitalizations (in 6.6% of hospitalizations, the patient died).

**Table 2:** Proportion of in-hospital all-cause deaths after admissions due to venous thromboembolism according to geographical region from 2008 to 2022 in Brazil's public health system.

Geographical region	Admissions	In-hospital deaths	
		n	%
North	16,469	970	5.89
Northeast	98,453	6,500	6.60
Center-West	42,761	2,234	5.22
Southeast	381,758	20,015	5.24
South	160,874	7,023	4.37
Total	700,315	36,742	5.25

## DISCUSSION

Throughout the last decades, several VTE registries have helped provide data on epidemiology, treatment modalities, risk factors, and outcomes^9^. The Registro Informatizado Enfermedad TromboEmbolica (RIETE) has enrolled patients with objectively confirmed VTE since 2001^10^. Other large-scale registries include the Venous Thrombosis Registry in Østfold Hospital (TROLL registry), which has enrolled consecutive patients diagnosed with, treated, and/or followed up for VTE at Østfold Hospital, Norway, since 2005; the Global Anticoagulant Registry in the FIELD-Venous Thromboembolic Events (GARFIELD-VTE)^11^; international or multicentric databases, such as the PREFER^12^ and the COMMAND VTE Registries^13,14^; and even registries of special subgroups such as the MASTER registry, which has provided evidence on the risk of VTE in patients undergoing mastectomy and immediate breast reconstruction^15^.

However, despite the continental proportions and vast population, no records of a standardized, large-scale registry of DVT cases have been reported in Brazil. In this study, we provide, to the best of our knowledge, the first account of a national survey of DVT hospitalizations, including an analysis of publicly available hard data, such as mortality.

Although the actual incidence of VTE and DVT remains elusive^16^, some authors report that at least 1–2 people per 1,000 population are affected by DVT or PE each year^17^. In contrast, others estimate that approximately 900,000 new cases of DVT occur in the United States annually, with a mortality rate close to 300,000 cases per year^18^.

The yearly average incidence of DVT in patients exclusively covered by the public health system in Brazil was comparable to those estimations. Nevertheless, it is important to note that this estimation is plausibly disparaged because nearly 30% of the population is privately insured and may not be represented in this sample.

The increased incidence in older individuals is already a factor described in the literature^6^.

It is interesting to note that, even though the Northeast region of Brazil concentrates around 30% of the country's population (according to the 2022 population census), only around 14% of hospitalizations due to VTE were recorded in this region. On the other hand, while the South region concentrates less than 15% of Brazil's population, it was responsible for registering more than 23% of thromboembolic events in the 15 years analyzed. The hospitalization rates for thrombosis per 100,000 inhabitants per year in the South region are much higher than those in the Northeast region.

It is well known that access to healthcare services is highly variable in Brazil^19^. The North and Northeast regions, known to be poorer, have greater difficulty accessing diagnostic and therapeutic services. In addition, it is not uncommon for individuals from these regions to migrate to wealthier ones in search of healthcare services^20,21^. In the South region, on the other hand, there is a greater density of healthcare services and more accessible territorial access, without the need to travel long distances, for example^22^. This may help explain these differences.

Another possible explanation (apart from patient migration) could be underdiagnosis in regions with less access to healthcare services. Lower socioeconomic status is associated with an increased risk of VTE in population studies^23,24^. One would expect a higher rate of hospitalizations in regions with lower per capita income. The low rates in the North and Northeast regions may mean that many cases of thrombosis are going unnoticed in these regions.

In terms of mortality from hospitalizations, on the other hand, the highest rates are found in the poorest regions. This is also supported by the literature, which shows that socioeconomically disadvantaged individuals tend to have higher rates of readmission and mortality associated with thromboembolic events^25^.

This study is limited mainly by the natural aspects of retrospective, big-data designs. First, the fact that the database is de-identified precludes a detailed analysis of patient demographics, risk factors, treatment modalities, and outcomes. The sample is substantially subject to coding errors, especially considering that the database is primarily administrative-focused instead of a clinical or research registry. Furthermore, due to the limitations of the database, it is not possible to say that in-hospital deaths occurred as a result of the thromboembolic event itself. We therefore analyzed all-cause mortality.

Finally, it is important to emphasize that the overall incidence may be underestimated due to a number of factors: first of all, it was also impossible to include in-hospital DVT cases that were not documented as admission diagnoses, such as cases of postoperative VTE. In addition, it is important to highlight that there is no organized record of cases diagnosed by private healthcare services, so it is impossible to define the incidence of cases in this population, which is known to have easier and quicker access to healthcare services and therefore tends to have a higher incidence of the diagnosis of thromboembolic events.

Despite its limitations, this is, to the best of our knowledge, the largest, if not the only, study encompassing 14 years of data on DVT admissions in the entire territory covered by the Brazilian public health system and provides new and unique insights into this disease's incidence, geographic distribution, cost, and mortality in the country.

## CONCLUSION

A total of 700,315 hospitalizations for VTE occurred between 2008 and 2022 in Brazil's public health system, with a yearly average of 46,688 cases, a total of 3.02 cases per 10,000 inhabitants.

The highest admission rates were registered in wealthier regions, while a higher proportion of deaths was found in the poorer ones.
